# Supplementary material for: Uncertainty in projections of future lake thermal dynamics is differentially driven by lake and global climate models
Source: PeerJ. 2023 Jun 2;11:e15445. doi: 10.7717/peerj.15445 (PMC10241169; doi:10.7717/peerj.15445)
Supplement: Supplemental Information 1 [file peerj-11-15445-s001.docx]

**Supplemental Information *for* Uncertainty in projections of future lake thermal dynamics is differentially driven by lake and global climate models**

Submitted as a Research Article to *PeerJ*

Jacob H. Wynne^1,2^, 0000-0003-4221-1247

Whitney M. Woelmer^1*^, 0000-0001-5147-3877

Tadhg N. Moore^1,3^, 0000-0002-3834-8868

R. Quinn Thomas^1,4^, 0000-0003-1282-7825

Kathleen C. Weathers^5^, 0000-0002-3575-6508

Cayelan C. Carey^1^, 0000-0001-8835-4476

^*^Corresponding author: [wwoelmer@vt.edu](mailto:wwoelmer@vt.edu)

**This SI contains:**

**Tables: 4**

**Figures: 13**

**References: 9**

**Pages: 20**

^1^Department of Biological Sciences, Virginia Tech, Blacksburg, Virginia, USA

^2^Current address: Department of Microbiology, Oregon State University, Corvallis, Oregon, USA

^3^Current address: School of Biological, Earth and Environmental Sciences, University College Cork, Cork, Ireland

^4^Department of Forest Resources and Environmental Conservation, Virginia Tech, Blacksburg, Virginia, USA

^5^Cary Institute of Ecosystem Studies, Millbrook, New York, USA

| **Table S1.** Description of the representative concentration pathways (RCPs) used in this study. See van Vuuren et al. (2011) for a detailed overview of all RCPs. | |
| --- | --- |
| **Name** | **Atmospheric Forcing** |
| RCP 2.6 | Peak radiative forcing at 3 W/m^2^ (490 ppm CO_2_) declining to 2.6 W/m^2^ by 2099 |
| RCP 6.0 | Peak radiative forcing stabilizing at 6 W/m^2^ (850 ppm CO_2_) by 2099 |
| RCP 8.5 | Continuous increase in radiative forcing at 8.5 W/m^2^ by 2099 (1370 ppm CO_2_) |

| **Table S2.** List of parameters in each model that were selected for calibration due to previous studies. Parameter ranges were selected following Moore et al. (2021) and are reported below. All parameters were bounded by their respective ranges and optimized by a Latin Hypercube simulation of 500 iterations. | | | | | |
| --- | --- | --- | --- | --- | --- |
| **Model** | **Parameters** | **Description of parameter function** | **Ranges sampled** | **Final calibrated values** | **References** |
| FLake | wind_speed | Wind speed scaling factor | 0.5-1.5 | 1.4686 | (Bernhardt et al., 2012; Layden et al., 2016; Salgado & Moigne, 2010) |
|  | swr | Short wave radiation scaling factor | 0.5-1.5 | 0.7919 |  |
|  | c_relax_C | Constant in the relaxation equation for the shape factor | 0.0001-0.01 | 0.0009851 |  |
|  | depth_bs_lk | Depth of the thermally active layer of the bottom sediments | 0.1-6.0 | 0.37232 |  |
|  | T_bs_lk | Temperature at the outer edge of the thermally active layer of the bottom sediments | 4-20 | 18.555 |  |
| GLM | wind_speed | Wind speed scaling factor | 0.5-1.5 | 1.4167 | (Bruce et al., 2018; Bueche et al., 2017; Hipsey et al., 2019) |
|  | swr | Short wave radiation scaling factor | 0.5-1.5 | 0.89851 |  |
|  | sed_temp_mean (lower) | Annual mean sediment temperature | 0.4-20 | 6.7002 |  |
|  | sed_temp_mean (upper) | Annual mean sediment temperature | 0.4-20 | 11.854 |  |
|  | max_layer_thick | Maximum thickness of a layer | 0.2-0.75 | 0.21837 |  |
| GOTM | wind_speed | Wind speed scaling factor | 0.5-1.5 | 0.7952 | (Ayala et al., 2020) |
|  | swr | Short wave radiation scaling factor | 0.5-1.5 | 1.4135 |  |
|  | k_min | Minimum turbulent kinetic energy | 1.0e-12-1e-5 | 2.6303e-06 |  |
| Simstrat | wind_speed | Wind speed scaling factor | 0.5-1.5 | 1.4684 | (Gaudard et al., 2019; Peeters et al., 2002) |
|  | swr | Short wave radiation scaling factor | 0.5-1.5 | 0.93668 |  |
|  | a_seiche | Fraction of total wind energy which goes into seiche energy | 0.0001-0.01 | 0.0042714 |  |
| MyLake | wind_speed | Wind speed scaling factor | 0.5-1.5 | 1.4255 | (Saloranta & Andersen, 2007) |
|  | swr | Short wave radiation scaling factor | 0.5-1.5 | 1.2848 |  |
|  | C_shelter | Wind sheltering coefficient | 0.05-0.4 | 0.36905 |  |

| **Table S3**. Root mean square error (RMSE) of the water temperature of the whole water column (*T_WWC_* ), mean surface temperature (surface temperature), mean bottom temperature (bottom temperature), Schmidt stability, summer thermocline depth, summer stratification duration, and ice-off date for each lake model, and then the ensemble mean. Throughout all model simulations, goodness-of-fit calculations for FLake were made using the mean water column depth only (see *Materials & Methods: Lake Model Calibration and Validation*). We calculated these goodness-of-fit metrics separately for the calibration period (Cal; 2005-2015) and validation period (Val; 2015-2020). | | | | | | | | | | | | | | |
| --- | --- | --- | --- | --- | --- | --- | --- | --- | --- | --- | --- | --- | --- | --- |
| **Model** | ***T_WWC_Mean*(°C)** | | ***Surface Temperature* (°C)** | | ***Bottom Temperature***  **(°C)** | | ***Schmidt stability (J/m^2^)*** | | ***Thermocline Depth (m)*** | | ***Stratification Duration (days)*** | | ***Ice Off (days)*** | |
|  | *Cal* | *Val* | *Cal*  *n = 900* | *Val*  *n = 89* | *Cal*  *n = 20* | *Val*  *n = 18* | *Cal*  *n = 1447* | *Val*  *n = 603* | *Cal*  *n = 449* | *Val*  *n = 287* | *Cal*  *n = 7* | *Val*  *n = 7* | *Cal*  *n = 8* | *Val*  *n = 4* |
| FLake | 2.23 | 2.44 | 1.51 | 1.72 | NA | NA | 262.00 | 305.73 | 2.54 | 2.08 | 34.48 | 19.56 | 6.21 | 6.18 |
| GOTM | 1.91 | 2.60 | 1.13 | 1.57 | 4.69 | 5.67 | 74.77 | 95.70 | 4.01 | 4.25 | 43.09 | 48.39 | 55.14 | 70.46 |
| Simstrat | 1.48 | 2.27 | 1.03 | 1.01 | 4.03 | 7.29 | 46.36 | 91.08 | 2.74 | 2.76 | 33.14 | 23.74 | 4.17 | 28.58 |
| MyLake | 1.52 | 1.94 | 1.13 | 1.82 | 3.28 | 4.87 | 59.78 | 125.75 | 3.00 | 8.93 | 50.97 | 83.75 | 5.28 | 8.56 |
| GLM | 1.67 | 2.07 | 1.47 | 1.34 | 2.52 | 1.72 | 72.53 | 113.14 | 6.15 | 6.13 | 75.24 | 71.79 | NA | NA |
| Ensemble Mean | 1.29 | 1.69 | 0.85 | 0.76 | 3.40 | 4.74 | 71.04 | 95.85 | 1.65 | 2.18 | 41.06 | 43.61 | 13.20 | 26.37 |

| **Table S4**. Bias of the water temperature of the whole water column (*T_WWC_* ), mean surface temperature (surface temperature), mean bottom temperature (bottom temperature), Schmidt stability, summer thermocline depth, summer stratification duration, and ice-off date for each lake model, and then the ensemble mean. Throughout all model simulations, goodness-of-fit calculations for FLake were made using the mean water column depth only (see *Materials & Methods: Lake Model Calibration and Validation*). Summer is defined as June-August. We calculated these goodness-of-fit metrics separately for the calibration period (Cal; 2005-2015) and validation period (Val; 2015-2020). | | | | | | | | | | | | | | |
| --- | --- | --- | --- | --- | --- | --- | --- | --- | --- | --- | --- | --- | --- | --- |
| **Model** | ***T_WWC_Mean*(°C)** | | ***Surface Temperature, 1.0 m* (°C)** | | ***Bottom Temperature, 30.0 m* (°C)** | | ***Schmidt stability (J/m^2^)*** | | ***Thermocline Depth (m)*** | | ***Stratification Duration (days)*** | | ***Ice Off (days)*** | |
|  | *Cal* | *Val* | *Cal*  *n = 900* | *Val*  *n = 89* | *Cal*  *n = 20* | *Val*  *n = 18* | *Cal*  *n = 1447* | *Val*  *n = 603* | *Cal*  *n = 449* | *Val*  *n = 287* | *Cal*  *n = 7* | *Val*  *n = 7* | *Cal*  *n = 8* | *Val*  *n = 4* |
| FLake | -0.09 | -0.23 | 0.57 | 1.12 | NA | NA | 178.37 | 239.54 | -1.93 | -1.55 | 12.43 | 10.0 | -3.63 | 0.25 |
| GOTM | -0.53 | -0.82 | 0.32 | -0.94 | -3.43 | -4.89 | 36.02 | -21.81 | 3.60 | 4.06 | -35.29 | -46.50 | 54.88 | 68.25 |
| Simstrat | -0.09 | 0.85 | 0.05 | -0.77 | -2.98 | -6.89 | -9.69 | -1.19 | 2.14 | 2.43 | -25.71 | -18.75 | 1.13 | 24.25 |
| MyLake | 0.15 | 0.50 | -0.35 | -1.34 | -1.36 | -4.12 | -29.24 | -80.22 | 2.51 | -1.35 | -45.29 | -82.50 | -1.13 | 7.25 |
| GLM | -0.29 | -0.79 | -0.01 | 1.07 | -0.35 | -0.95 | -32.70 | -69.41 | -5.77 | -5.58 | -71.14 | -69.75 | NA | NA |
| Ensemble Mean | -0.15 | -0.04 | 0.11 | -0.17 | -2.04 | -4.21 | 28.55 | 13.38 | 0.11 | -0.39 | -33.11 | -41.50 | 12.81 | 25.00 |

*
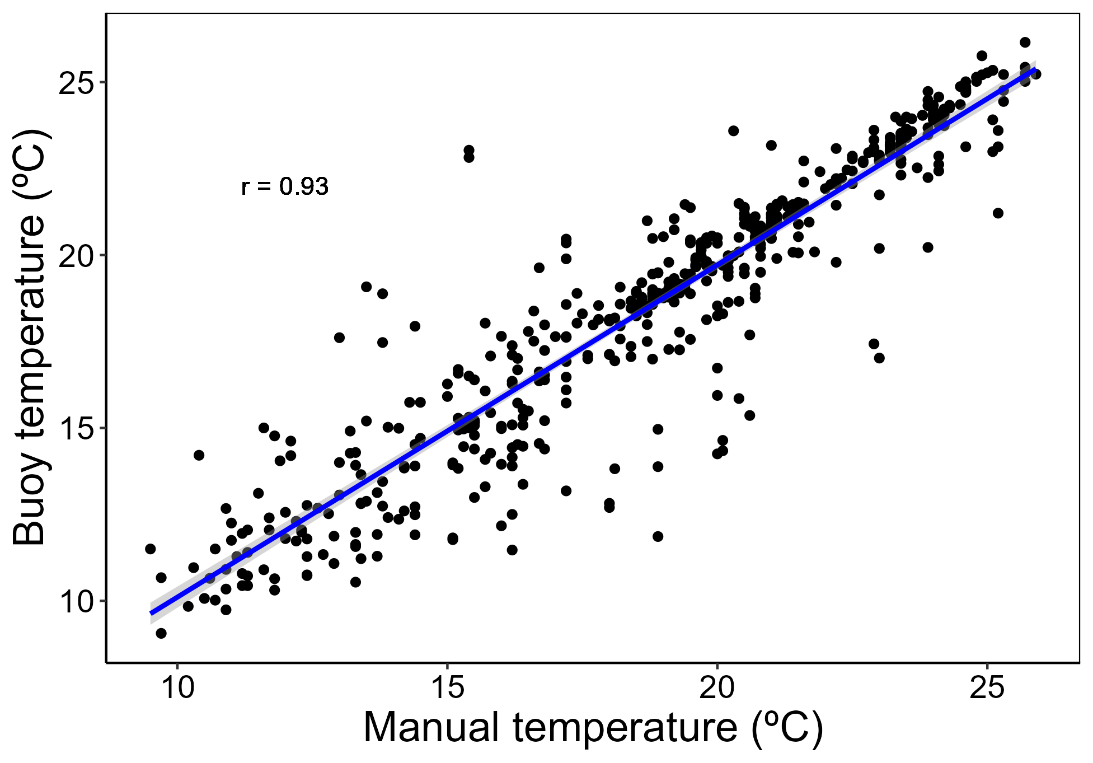
*

**Figure S1**. Comparison of paired water temperature observations from the surface to 10.0 m at Lake Sunapee, NH, USA from the buoy site near Loon Island Lighthouse (43.391°N, 72.058°W) and long-term manual sampling site (43.3844°N, 72.0624°W). A Pearson correlation yielded highly-significant comparison between the sites (r=0.93; p<0.0001; n=557).


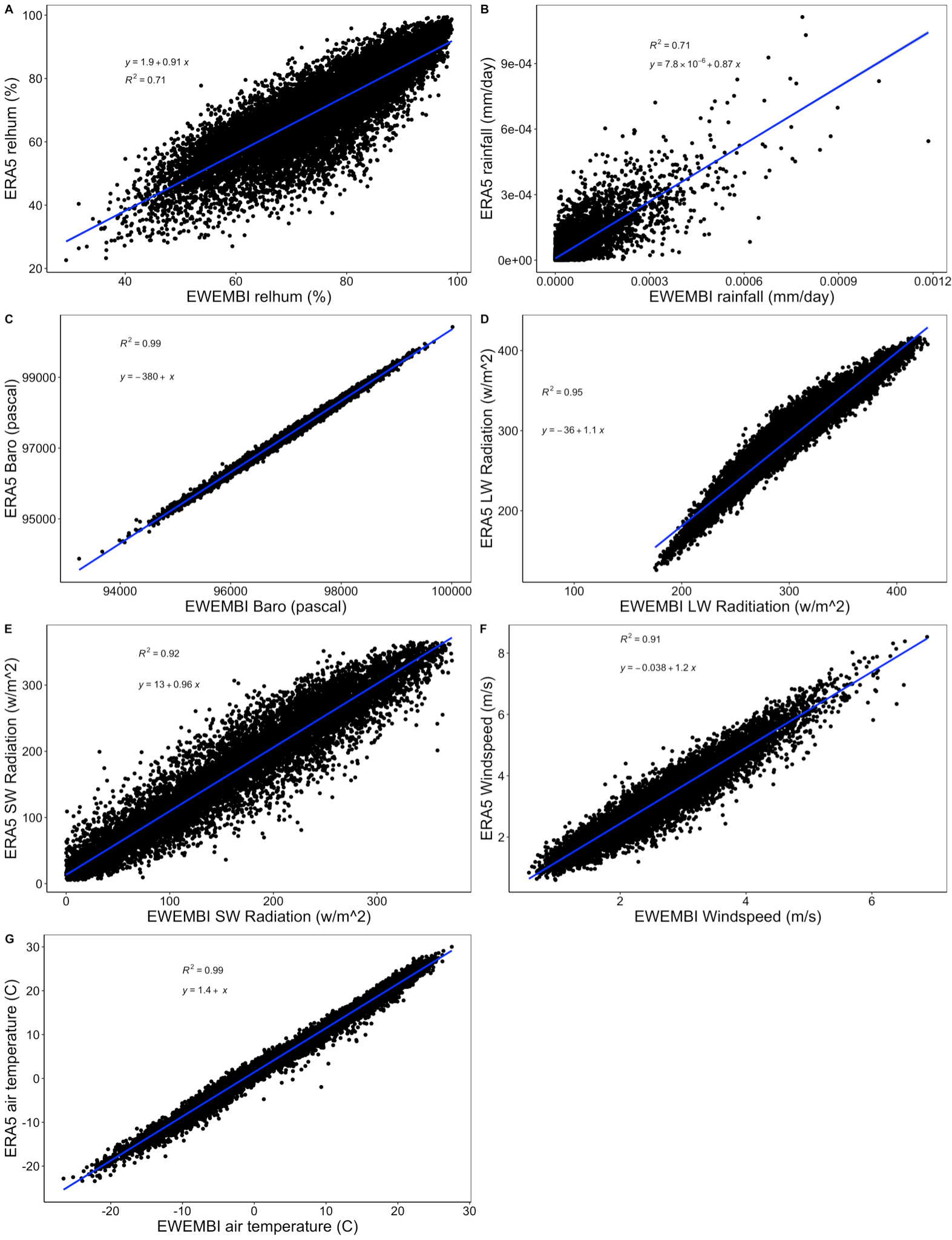


**Figure S2.** Comparisons between EWEMBI (EartH2Observe, WFDEI and ERA-Interim data Merged and Bias-corrected for ISIMIP) and ERA5 modeled meteorological data for Lake Sunapee during 1979-2016 for A) relative humidity (%), B) total rainfall (mm/day), C) surface barometric pressure (pascal), D) longwave radiation (watt/m^2^), E) shortwave radiation (watt/m^2^), F) wind speed at 10.0m elevation (m/s), and G) air temperature (°C). The linear model and R^2^ goodness-of-fit from the comparison are presented within each panel.


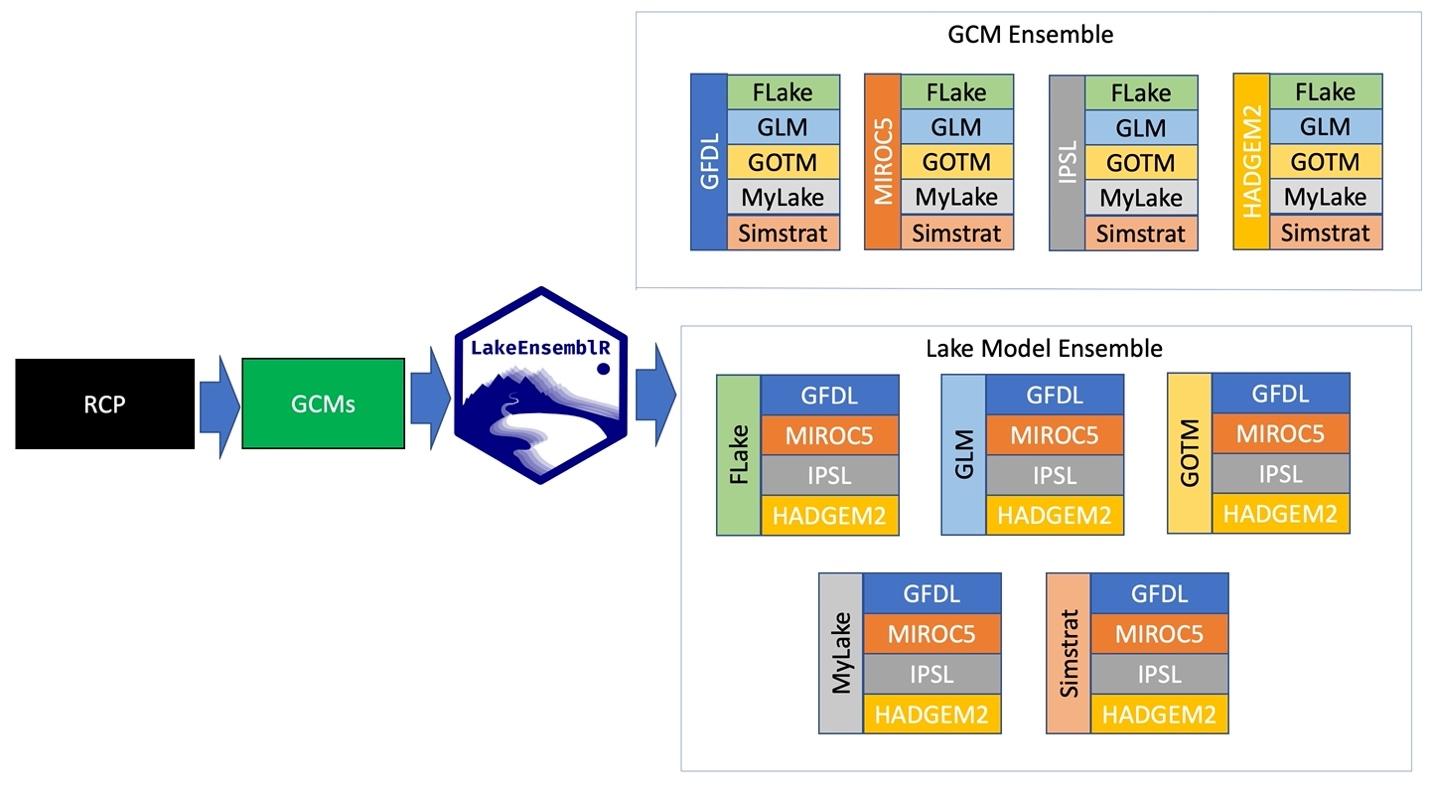


**Figure S3.** Conceptual figure showing the workflow for calculating ensemble model interactions within our ensemble projections for a single Representative Concentration Pathway (RCP) scenario. Each RCP was used to simulate all four General Circulation Models (GCMs), which provided the climate driver data to run all five lake models using the R LakeEnsemblR package. The right side of the figure shows the groupings for each GCM (top) or Lake Model (bottom) which were used to calculate ensemble means across each grouping, where each GCM and lake model is identified by a unique color (see *Materials & Methods: Ensemble model interactions*).


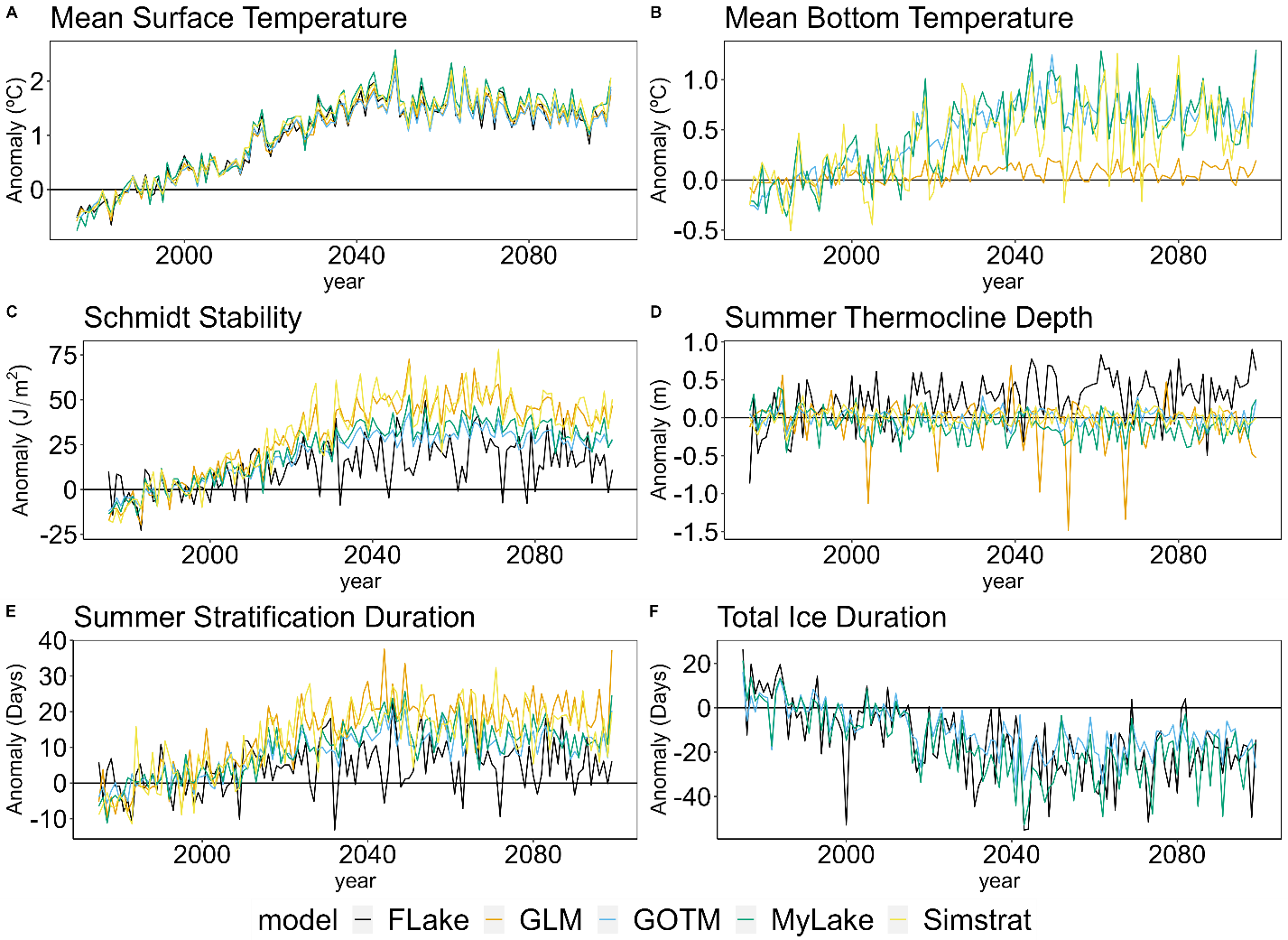


**Figure S4.** Projections for Lake Sunapee, NH, USA grouped by GCM for each lake model under RCP 2.6 for: A) mean surface temperature, B) mean bottom temperature, C) Schmidt stability, D) summer thermocline depth (June – August), E) summer stratification duration, and F) total ice duration from 2006-2099. These plots represent lake model uncertainty, as the variability due to GCMs was held constant for each lake model.


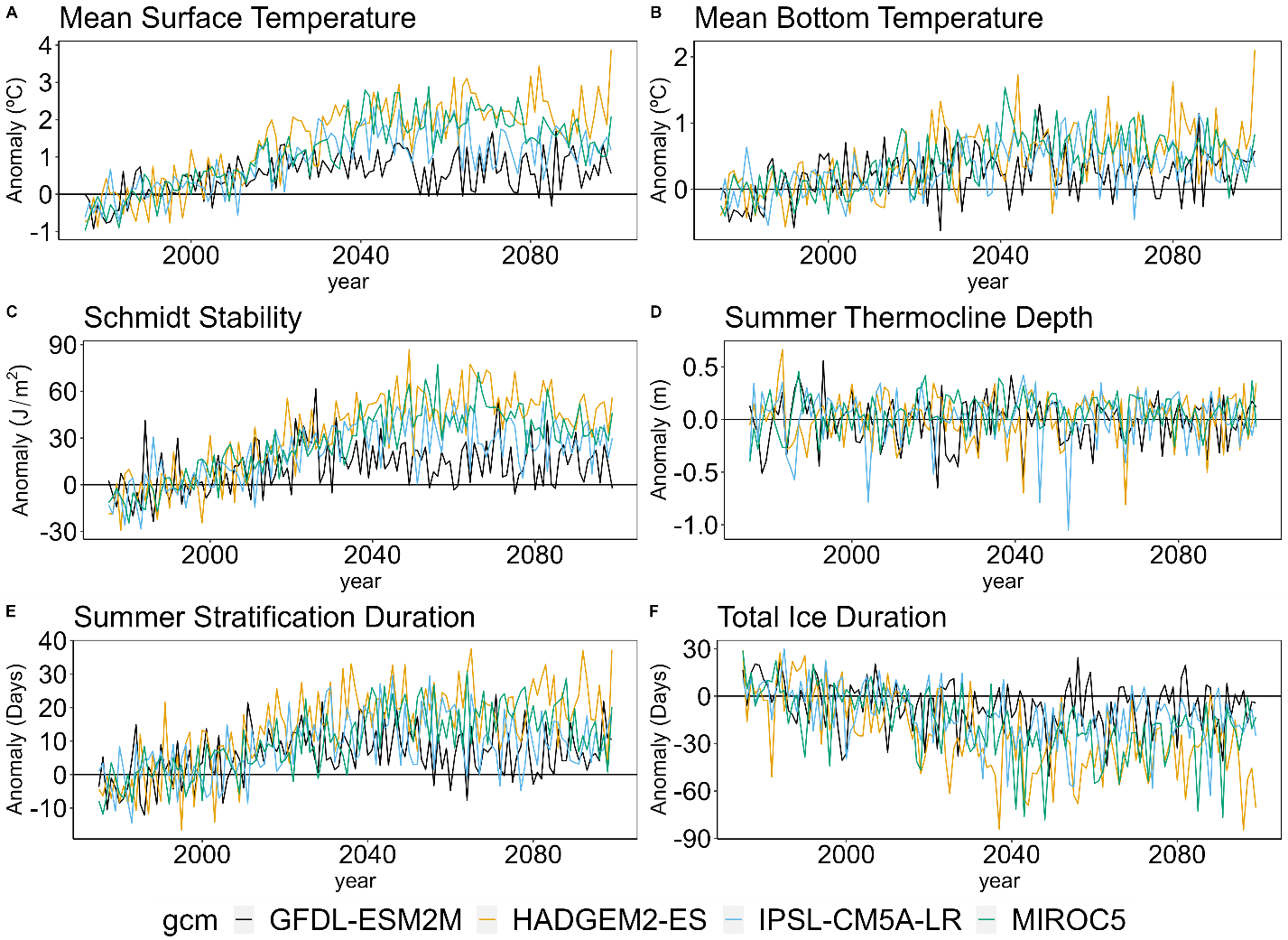


**Figure S5.** Projections at Lake Sunapee, NH, USA grouped by lake model for each GCM under RCP 2.6 for A) mean surface temperature, B) mean bottom temperature, C) Schmidt stability, D) summer thermocline depth (June – August), E) summer stratification duration, and F) total ice duration from 2006-2099. These plots represent GCM uncertainty, as the variability due to lake model was held constant for each metric.


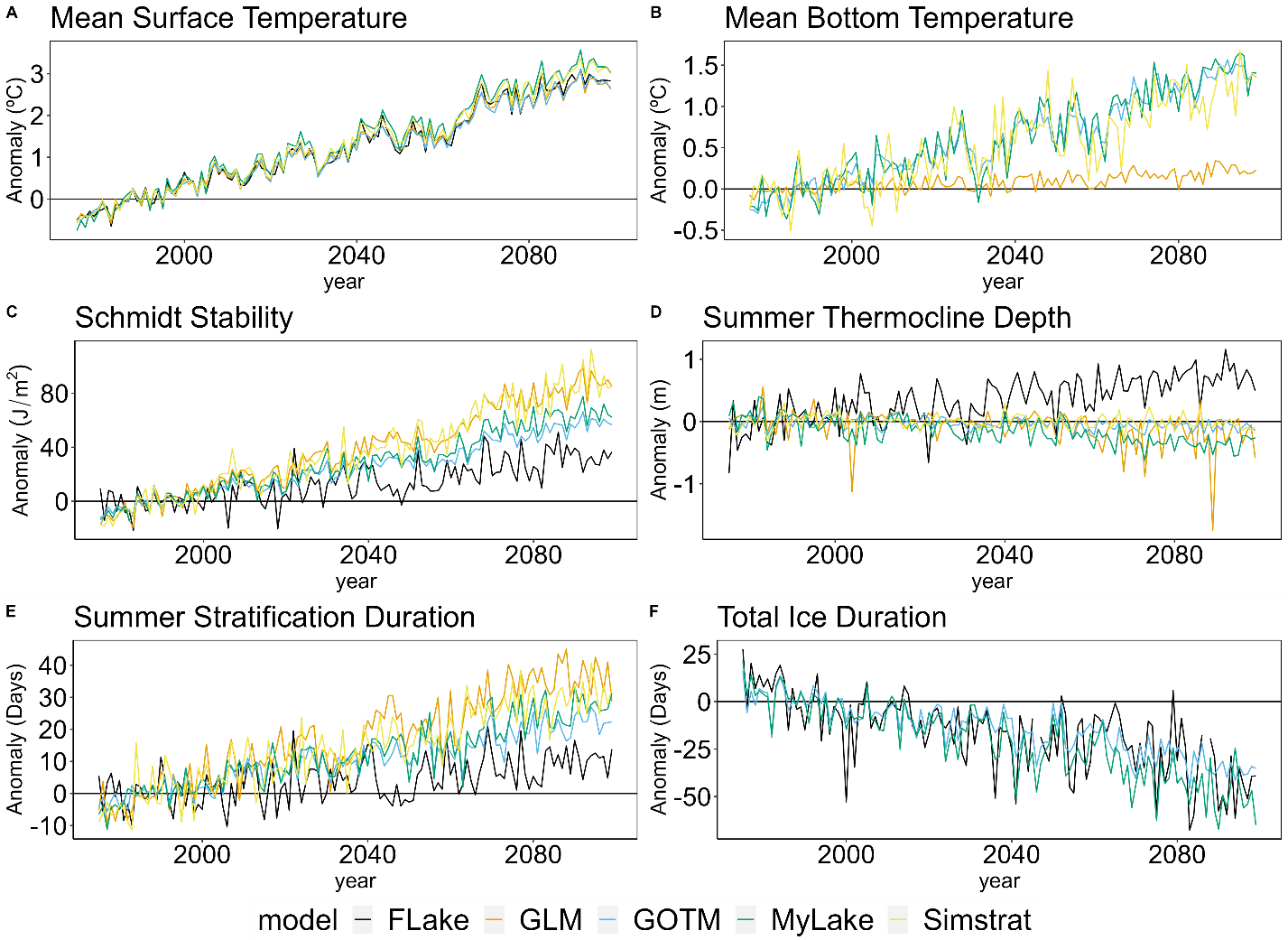


**Figure S6**. Projections at Lake Sunapee, NH, USA grouped by GCM for each lake model under RCP 6.0 for A) mean surface temperature, B) mean bottom temperature, C) Schmidt stability, D) summer thermocline depth (June – August), E) summer stratification duration, and F) total ice duration from 2006-2099. These plots represent lake model uncertainty, as the variability due to lake model was held constant for each GCM.


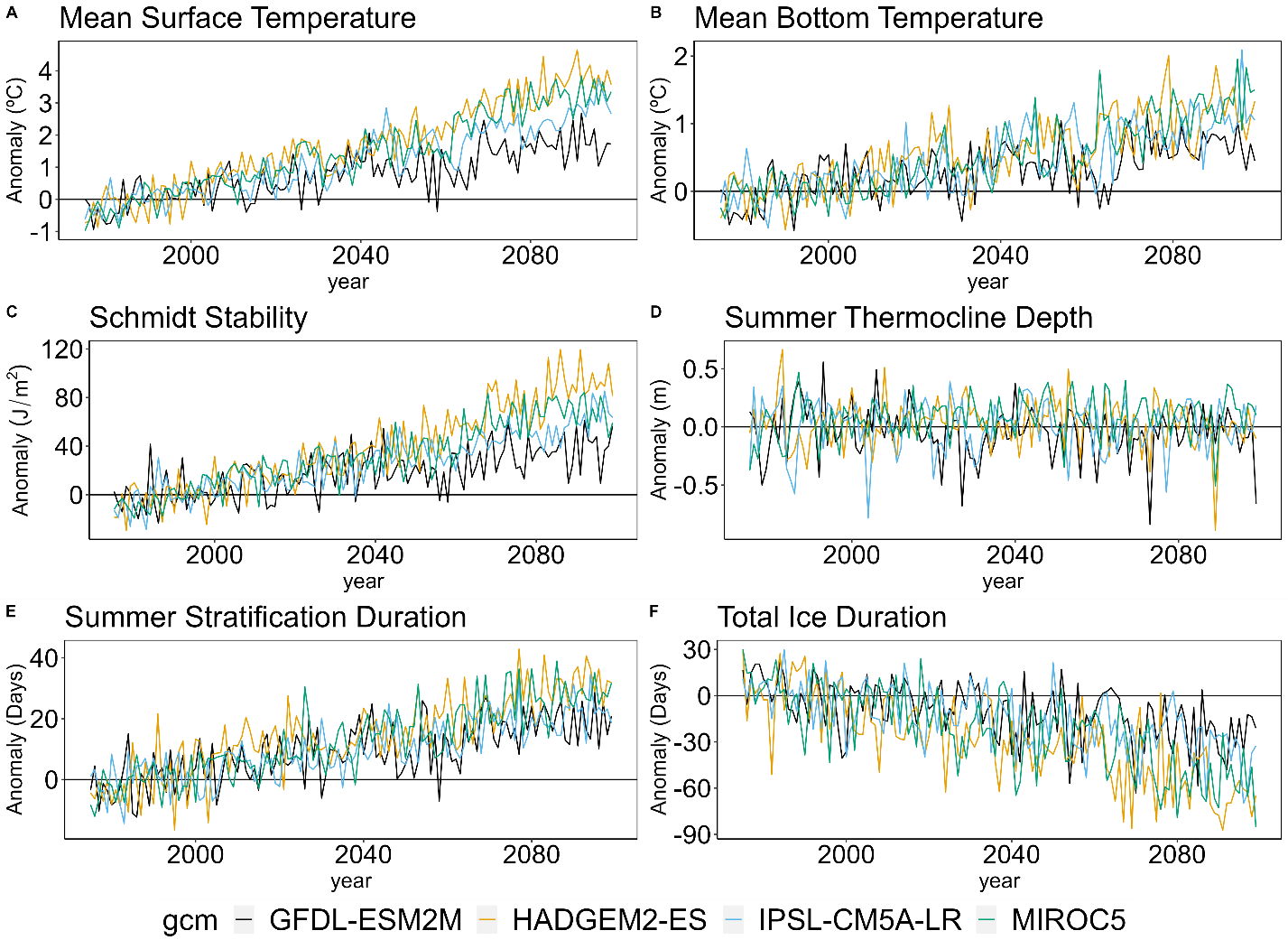


**Figure S7.** Projections at Lake Sunapee, NH, USA grouped by lake model for each general circulation model (GCM) under RCP 6.0 for A) mean surface temperature, B) mean bottom temperature, C) Schmidt stability, D) summer thermocline depth (June – August), E) summer stratification duration, and F) total ice duration from 2006-2099. These plots represent GCM uncertainty, as the variability due to lake model was held constant for each metric.


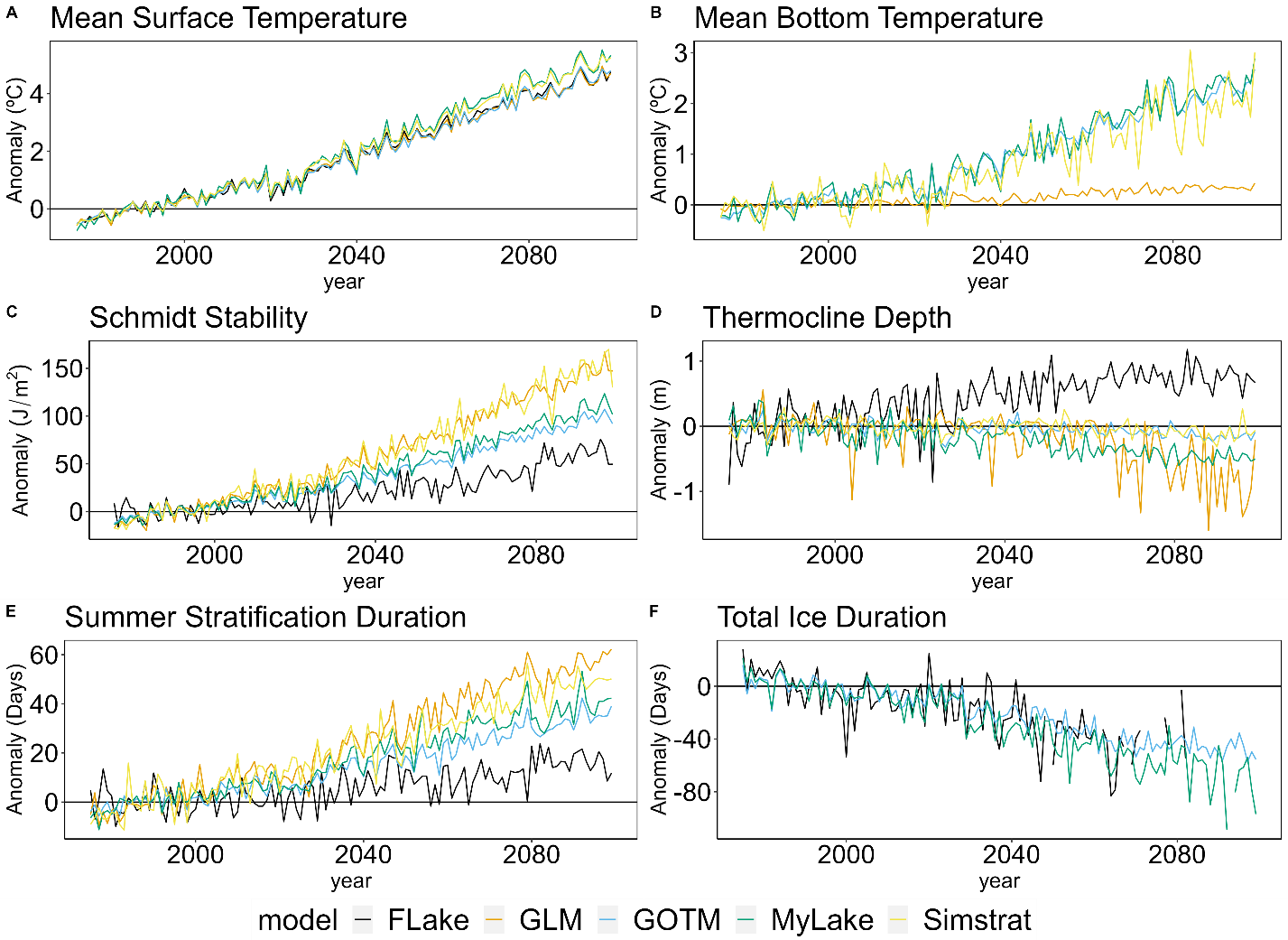


**Figure S8.** Projections at Lake Sunapee, NH, USA grouped by general circulation model (GCM) for each lake model under RCP 8.5 for A) mean surface temperature, B) mean bottom temperature, C) Schmidt stability, D) summer thermocline depth (June – August), E) summer stratification duration, and F) total ice duration from 2006-2099. These plots represent lake model uncertainty, as the variability due to GCM was held constant for each metric.


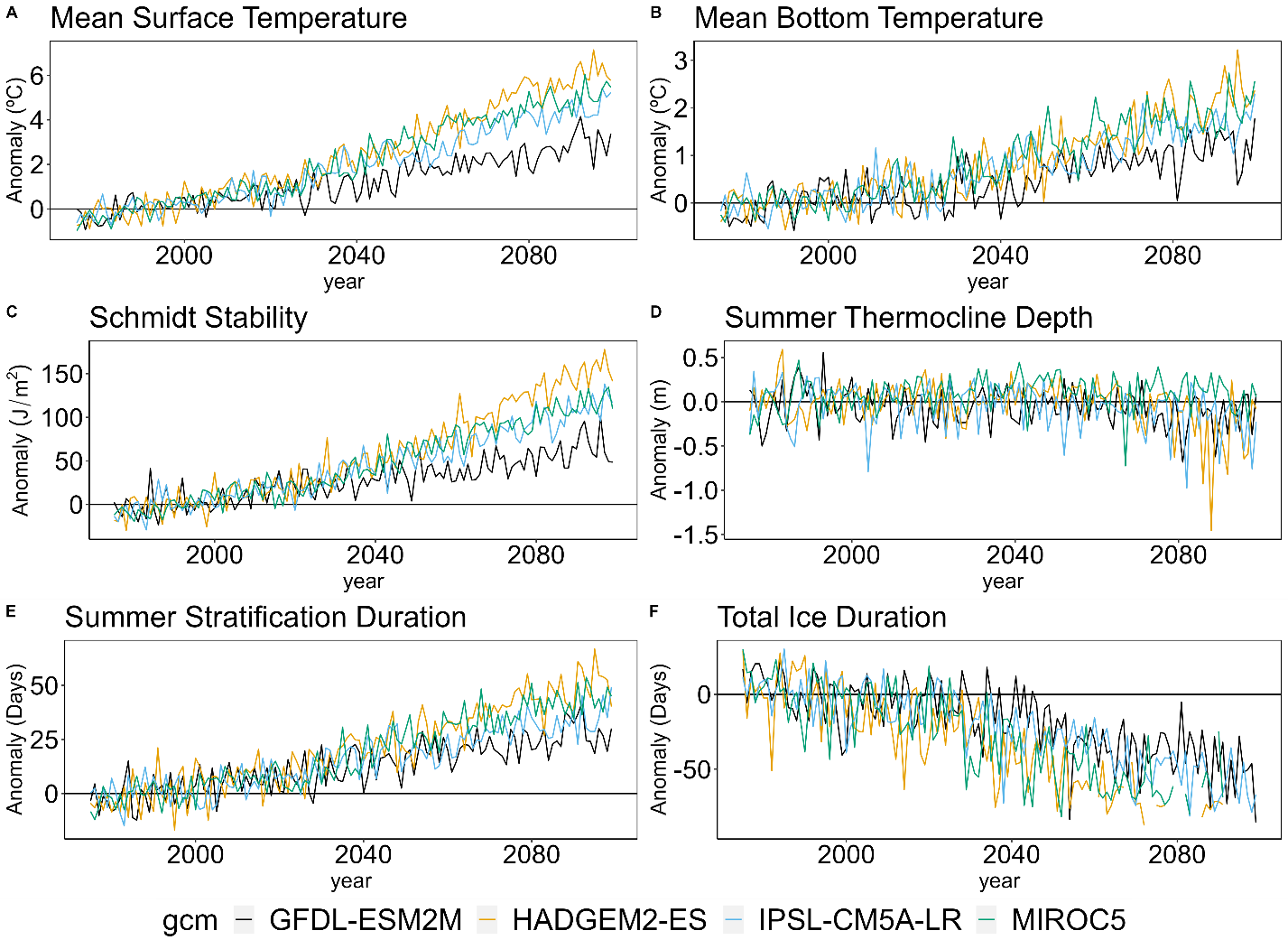


**Figure S9.** Projections at Lake Sunapee, NH, USA grouped by lake model for each general circulation model (GCM) under RCP 8.5 for A) mean surface temperature, B) mean bottom temperature, C) Schmidt stability, D) summer thermocline depth (June – August), E) summer stratification duration, and F) total ice duration from 2006-2099. These plots represent GCM uncertainty, as the variability due to lake model was held constant for each metric.


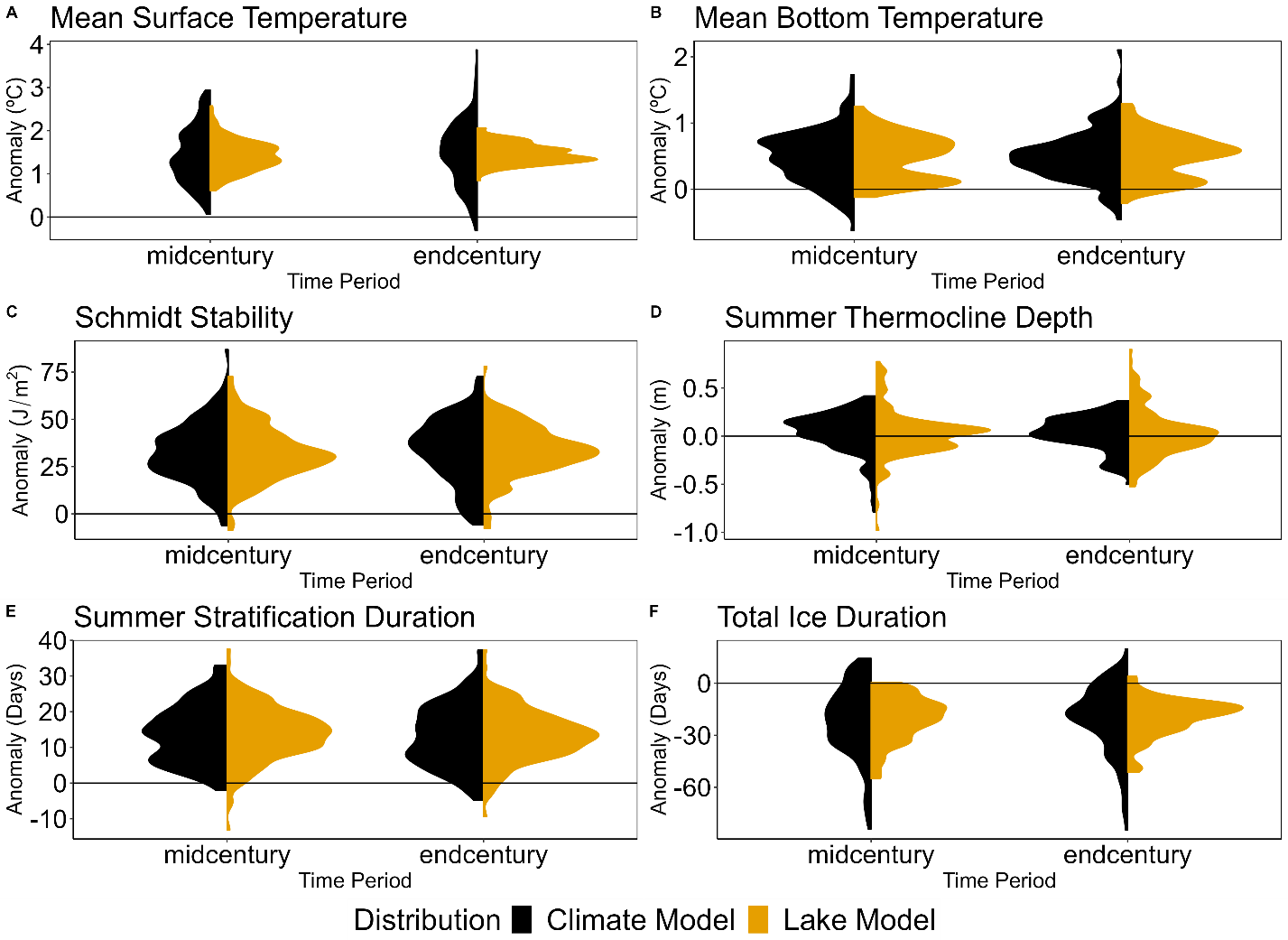


**Figure S10.** Ensemble distributions of the model-type ensemble means of the climate and lake models under RCP 2.6 at Lake Sunapee, NH, USA. These distributions were calculated for A) mean surface temperature, B) mean bottom temperature, C) Schmidt stability, D) summer thermocline depth (June – August), E) summer stratification duration, and F) total ice duration during mid-century (2020-2050) and end-century (2069-2099).


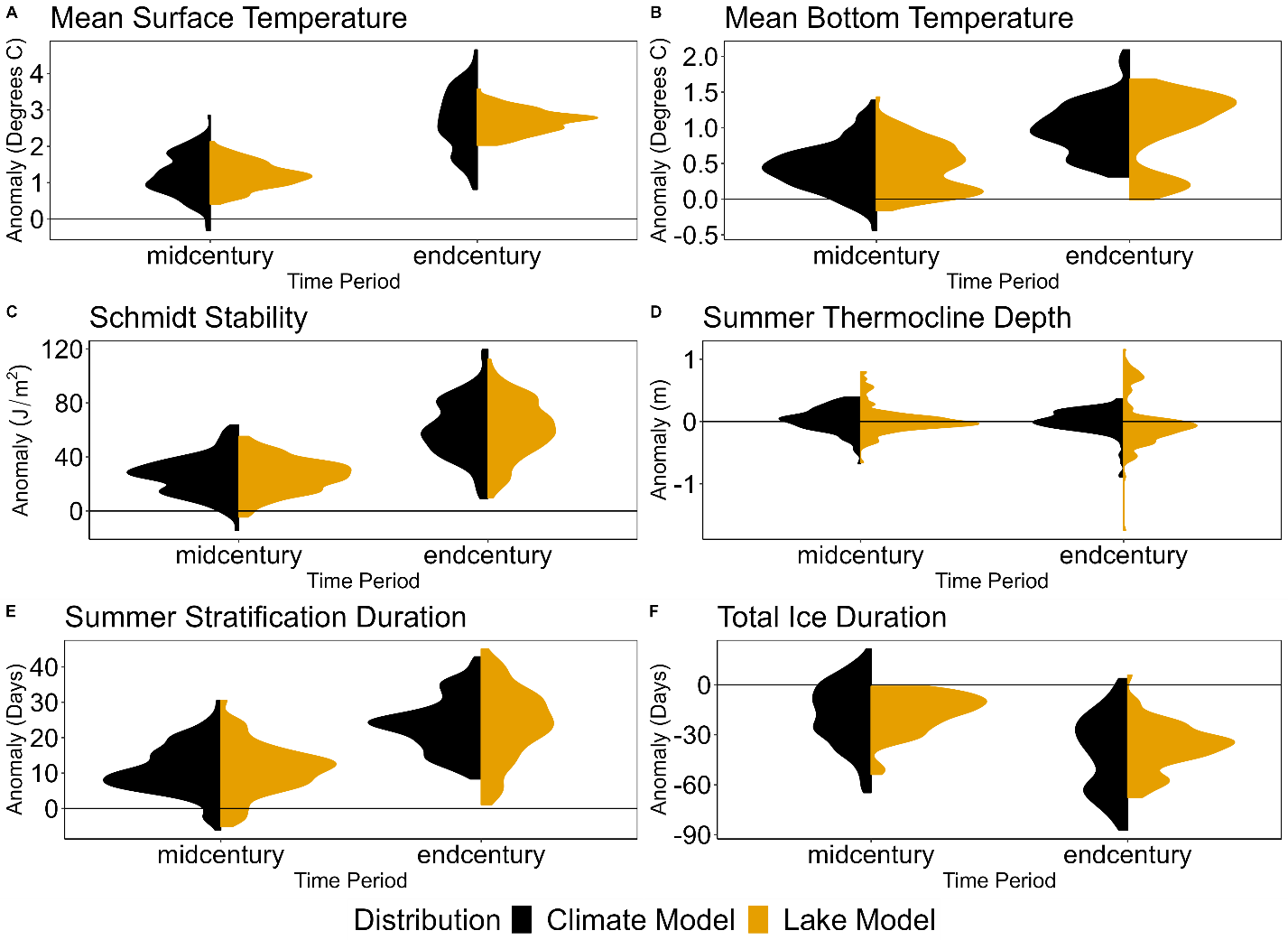


**Figure S11.** Ensemble distributions of the model-type ensemble means of the climate and lake models under RCP 6.0 at Lake Sunapee, NH, USA. These distributions were calculated for A) mean surface temperature, B) mean bottom temperature, C) Schmidt stability, D) summer thermocline depth (June – August), E) summer stratification duration, and F) total ice duration during mid-century (2020-2050) and end-century (2069-2099).


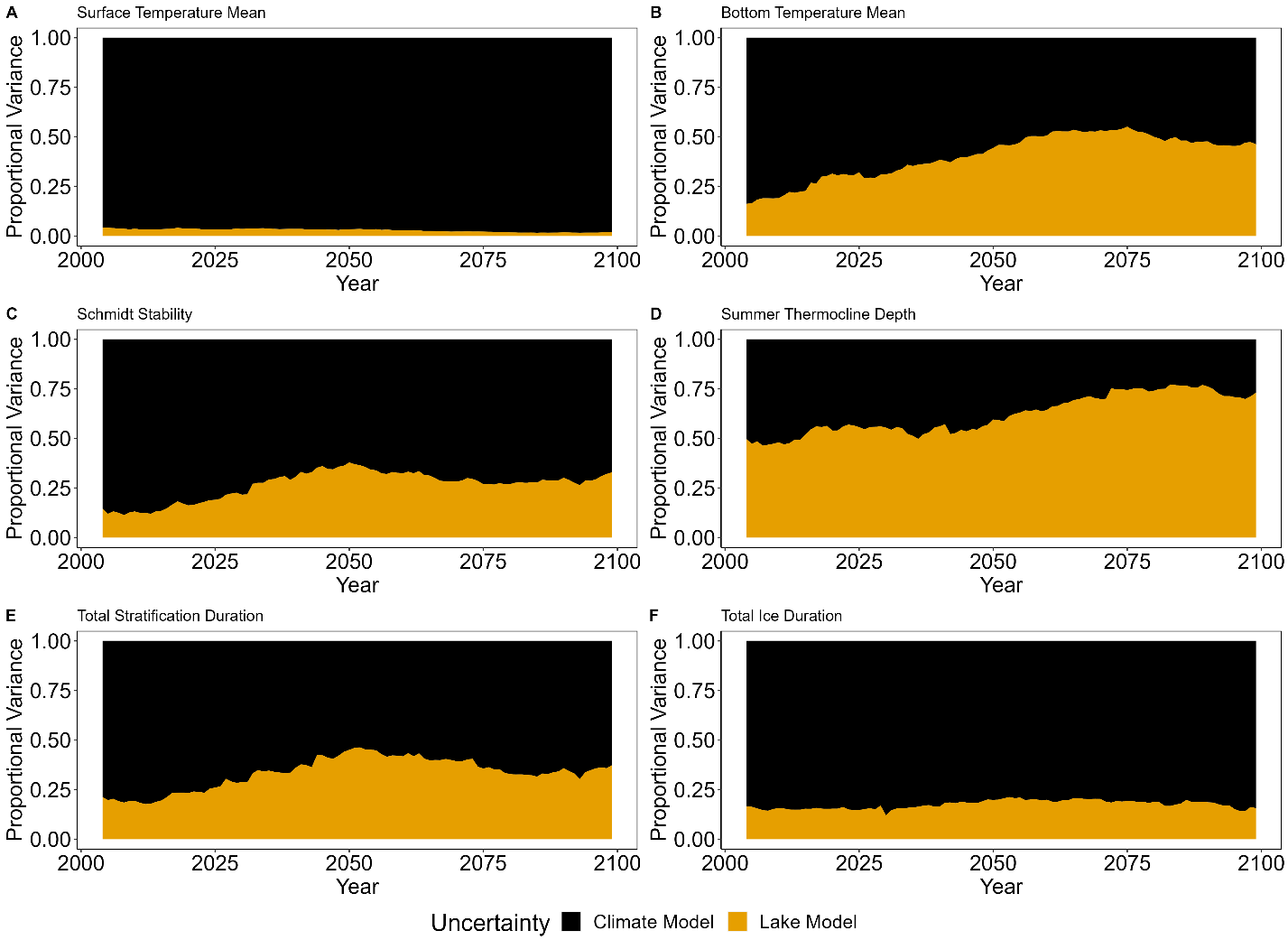


**Figure S12.** Proportional variance across projections at Lake Sunapee, NH, USA under RCP 2.6 for A) mean surface temperature, B) mean bottom temperature mean, C) Schmidt stability, D) summer thermocline depth (June – August), E) summer stratification duration, and F) total ice duration from 2006-2099.


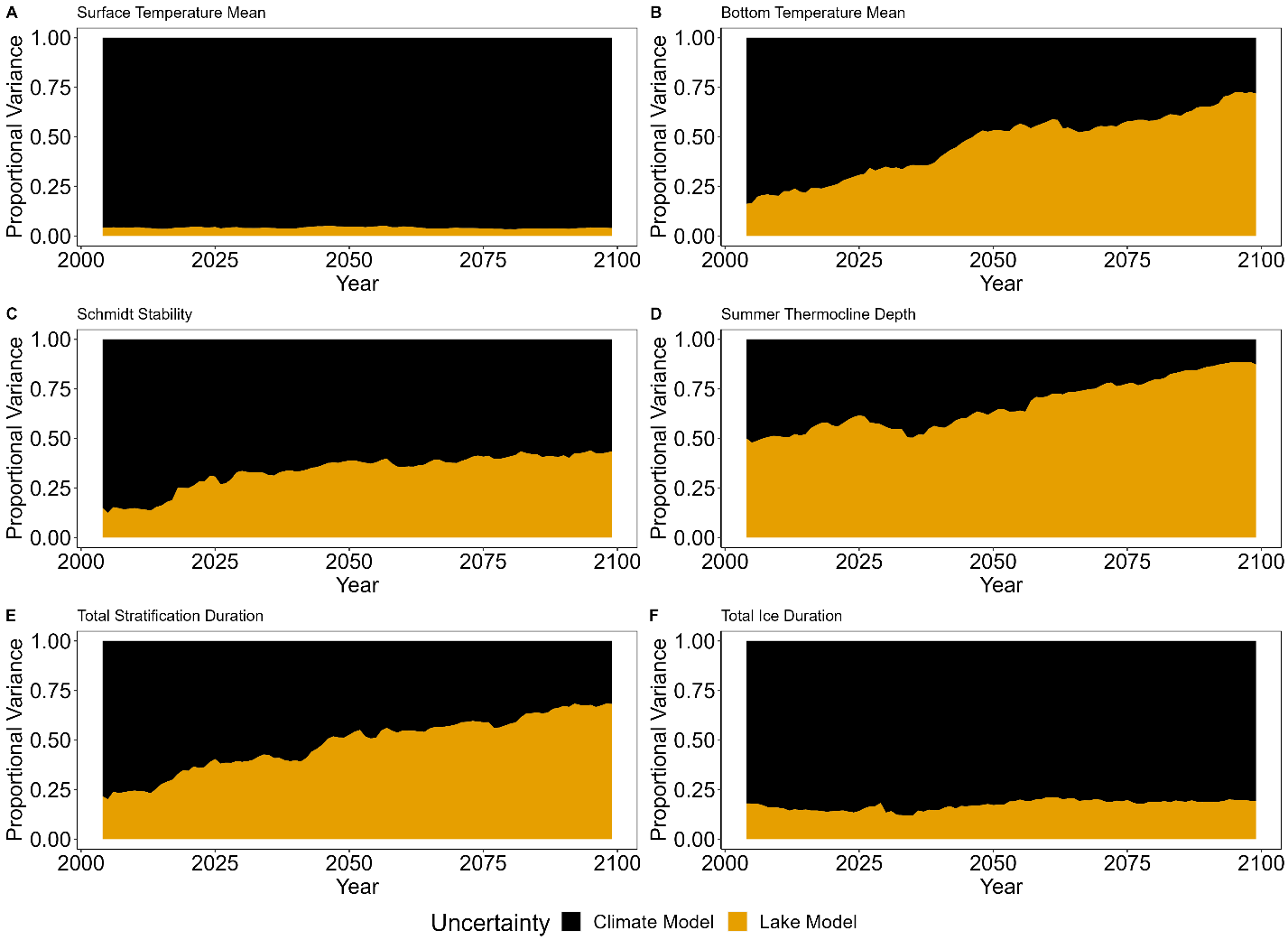


**Figure S13.** Proportional variance across projections at Lake Sunapee, NH, USA under RCP 6.0 for A) mean surface temperature, B) mean bottom temperature, C) Schmidt stability, D) summer thermocline depth (June – August), E) summer stratification duration, and F) total ice duration from 2006-2099.

**References**

Ayala AI, Moras S, Pierson DC. 2020. Simulations of future changes in thermal structure of Lake Erken: proof of concept for ISIMIP2b lake sector local simulation strategy. *Hydrology and Earth System Sciences* 24:3311–3330. DOI: 10.5194/hess-24-3311-2020.

Bernhardt, J, Engelhardt, C, Kirillin, G, & Matschullat, J. 2012. Lake ice phenology in

Berlin-Brandenburg from 1947–2007: observations and model hindcasts. Climatic Change, 112(3), 791-817. <https://doi.org/10.1007/s10584-011-0248-9>

Bruce, LC, Frassl, MA, Arhonditsis, GB, Gal, G, Hamilton, DP, Hanson, PC, Hetherington, AL, Melack, JM, Read, JS, Rinke, K, Rigosi, A, Trolle, D, Winslow, L, et al. 2018. A multi-lake comparative analysis of the General Lake Model (GLM): Stress-testing across a global observatory network. Environ. Model. Softw. 102, 274-291.

Bueche, T, Hamilton, DP, Vetter, M. 2017. Using the General Lake Model (GLM) to simulate water temperatures and ice cover of a medium-sized lake: a case study of Lake Ammersee, Germany. Environ. Earth Sci., 76: 461, [10.1007/s12665-017-6790-7](https://doi.org/10.1007/s12665-017-6790-7)

Gaudard, A, Råman Vinnå, L, Bärenbold, F, Schmid, M, Bouffard, D. 2019. Toward an open access to high-frequency lake modeling and statistics data for scientists and practitioners – the case of Swiss lakes using Simstrat v2.1. Geosci Model Dev, 12: 3955-3974, [10.5194/gmd-12-3955-2019](https://doi.org/10.5194/gmd-12-3955-2019)

Layden, A, MacCallum, SN, & Merchant, CJ. 2016. Determining lake surface water temperatures worldwide using a tuned one-dimensional lake model (FLake, v1). Geoscientific Model Development, 9(6), 2167-2189. https://doi.org/10.5194/gmd-9-2167-2016

Peeters, F, Livingstone, DM, Goudsmit, GH, Kipfer, R, & Forster, R. 2002. Modeling 50 years of historical temperature profiles in a large central European lake. Limnology and Oceanography. 47(1), 186-197. https://doi.org/10.4319/lo.2002.47.1.0186

Salgado, R & Le Moigne, P. 2010. Coupling of the FLake model to the Surfex externalized surface model. Boreal Environmental Research, 15, 231-244.

Saloranta, TM, Andersen, T. 2007. MyLake—a multi-year lake simulation model code suitable for uncertainty and sensitivity analysis simulations Ecol. Model., Uncertainty in Ecological Models, 207: 45-60, [10.1016/j.ecolmodel.2007.03.018](https://doi.org/10.1016/j.ecolmodel.2007.03.018)
